# The NUTRI-OBEDIGITHUM Pathway: Integrating Personalized Exchange-Based Nutrition into Digitally Enabled Obesity Care and a Retrospective Real-World Cohort Analysis

**DOI:** 10.3390/nu18182985

**Published:** 2026-09-11

**Authors:** Clotilde Vázquez, Jersy Cárdenas-Salas, Francisco Arrieta, Alvaro Sánchez, Mar Alcarria, Alicia Melchor, Bogdana Luca, Clara Yela, Yvonne Fernández, Ana Prieto, Amalia Paniagua, Maite Ortega, Leopoldo García-Valdecasas, Adriana Burrero, Alberto Martínez, Nerea Aguirre, Miguel Aganzo, Ana Isabel de Cos, Esmeralda Martín, Juan Gómez-Borrallo, Marbella Piñera, Roberto Sierra, Marta del Olmo, Sebastian Mas-Fontao, Teresa Montoya

**Affiliations:** 1 Department of Endocrinology and Nutrition, Hospital Universitario Fundación Jiménez Díaz, 28040 Madrid, Spain; jersy.cardenas@quironsalud.es (J.C.-S.);; 2 Instituto de Investigación Sanitaria Fundación Jiménez Díaz, 28040 Madrid, Spain; 3 Department of Endocrinology and Nutrition, Hospital Universitario Infanta Elena, 28342 Madrid, Spain; 4 Department of Endocrinology and Nutrition, Hospital Universitario Rey Juan Carlos, 28933 Madrid, Spain; 5 Department of Endocrinology and Nutrition, Hospital Universitario de Villalba, 28400 Madrid, Spain; 6 Comité de Ética Asistencial, Hospital Universitario La Paz, 28046 Madrid, Spain; 7 Department of Endocrinology and Nutrition, Hospital Universitario Severo Ochoa, 28914 Madrid, Spain; 8 Overweight & Obesity Institute, 28040 Madrid, Spain; 9 Primary Care Centre “Benita Ávila”, 28043 Madrid, Spain; 10 Corporate Management, Quirónsalud 4H, 28040 Madrid, Spain

**Keywords:** obesity, nutritional prescription, exchange-based diet, digital health, personalized nutrition, real-world data, implementation

## Abstract

Background/Objectives: Obesity care requires nutritional prescriptions that are individualized, nutritionally coherent, understandable, and feasible within high-volume clinical services. We describe the NUTRI-OBEDIGITHUM pathway, which integrates multidimensional digital phenotyping, clinician-supervised exchange-based nutritional prescription, patient-oriented materials, and structured education, and we report preliminary descriptive outcomes from a retrospective real-world cohort. Methods: This descriptive methodological and operational study included a retrospective observational analysis of anonymized routine-care data. NUTRI-OBEDIGITHUM integrates OBEDIGITHUM, a digitally enabled obesity-care pathway, with Dietcamb^®^, a software-supported method that translates individualized energy and macronutrient targets into food exchanges, meal distributions, household measures, food lists, and example menus. The source database comprised 3928 unique patients. Among patients with a plausible baseline weight and an observable 6-month window, the eligible outcome was the measurement closest to day 183 within ±60 days, after prespecified plausibility checks. Baseline characteristics were compared descriptively according to outcome availability. Baseline medication exposure was assessed using free-text mentions only. Results: By the data cutoff, the 6-month window had opened for 3215 patients; 929 (28.9%) had an eligible 6-month weight and 2286 (71.1%) did not. In the analyzed cohort, mean age was 53.7 ± 14.0 years, 70.5% were women, and mean body mass index was 35.9 ± 6.3 kg/m^2^. Mean weight changed from 98.5 ± 20.7 kg at baseline to 94.2 ± 20.3 kg at follow-up, corresponding to a descriptive paired change of −4.3 kg (95% CI −4.7 to −3.9); median total body-weight loss was 3.4%. Losses of at least 5%, 10%, and 15% were observed in 39.6%, 17.8%, and 6.4% of patients, respectively. A baseline GLP-1 receptor-agonist mention was present in 151/929 patients (16.3%); medication timing, dose, duration, dispensing, persistence, and adherence were unavailable. Conclusions: NUTRI-OBEDIGITHUM provides an operational framework for converting multidimensional obesity phenotyping into a structured, flexible, clinician-supervised, and patient-oriented nutritional prescription. The observed weight trajectories are descriptive complete-case findings from a selected, uncontrolled cohort and cannot establish the independent effectiveness of the pathway, Dietcamb^®^, or pharmacotherapy. Prospective studies should evaluate reach, adoption, fidelity, usability, adherence, nutritional adequacy, safety, NUTRI-specific workload and cost, and metabolic outcomes.

## 1. Introduction

Obesity is a chronic, heterogeneous, and progressive disease with major consequences for morbidity, quality of life, and healthcare sustainability, and its clinical severity cannot be defined by body mass index alone [1,2,3]. Lifestyle modification remains a cornerstone of treatment even in the era of effective anti-obesity pharmacotherapy, and its long-term effectiveness depends less on a single optimal dietary pattern than on the patient’s ability to understand, adapt, and maintain the prescription [4,5,6,7].

This challenge is particularly acute in Spain. General obesity affected 21.6% of adults aged 25–64 years in the ENPE study [8], and a more recent nationwide analysis estimated that 18.7% of Spanish adults have obesity and over half have excess weight [9]. Spain remains, moreover, the only European Union country without systematic incorporation of dietitians/nutritionists into its National Health System across most regions [10]; the World Health Organization recommended a ratio of one dietitian/nutritionist per 50 patients in regional and intermediate hospitals, a target that Spanish public hospitals have not met [11]. The Spanish ACTION-IO cohort further documented delayed conversations about weight and low rates of formal diagnosis [12]. In this setting, the number of patients requiring nutritional assessment routinely exceeds the capacity for repeated, individualized dietitian/nutritionist consultations, so that services often default to general advice or standardized diet plans rather than a prescription adapted to each patient’s phenotype.

An individualized nutritional prescription must integrate metabolic phenotype, body composition, comorbidities, pharmacological treatment, eating behavior, dietary restrictions, food preferences, and lifestyle constraints; translating this into a safe, coherent, and immediately actionable prescription within a single high-volume consultation is therefore a major operational bottleneck that current alternatives do not resolve [13,14,15]. Exchange-based dietary systems offer a structured way to translate energy and macronutrient targets into practical food portions while preserving flexibility in food selection [16], but their clinical integration requires more than an exchange table: the prescription must be linked to the patient’s phenotype, clinically supervised, and reinforced through continued education.

To address this gap, our group developed NUTRI-OBEDIGITHUM by integrating two original tools progressively refined through clinical practice: OBEDIGITHUM, a clinician-led, digitally enabled pathway for multidimensional obesity care, and Dietcamb^®^, a software-supported method for exchange-based nutritional prescription [17,18]. Dietcamb^®^ was conceived not as a stand-alone application or a fixed obesity diet, but as an adaptable method for translating individualized nutritional targets into food exchanges, meal distribution, household measures, food lists, and example menus [19,20]. The pathway is currently used across four hospitals within the Quirónsalud 4Hospitales network in Madrid, which collectively receive more than 2000 new patients with obesity each year.

The specific gap addressed here is operational: there is little empirical description of how multidimensional obesity phenotyping can be converted during routine high-volume care into an immediately usable, clinician-supervised exchange prescription, together with transparent denominators and preliminary real-world follow-up. The pathway was designed to make an individualized prescription available from the first specialist encounter and to support its practical use through structured group education. We therefore aimed (1) to describe the development and integration of the NUTRI-OBEDIGITHUM pathway; (2) to report its concrete clinical outputs and service-user-informed refinements; and (3) to characterize the selection, baseline features, medication recording, and weight trajectories of patients with an eligible 6-month measurement. We did not aim to estimate causal efficacy or conduct a formal implementation-effectiveness or usability evaluation.

## 2. Methods

### 2.1. Methodological Scope and the OBEDIGITHUM Pathway

This article provides a descriptive methodological and operational account of the NUTRI-OBEDIGITHUM pathway, a clinician-supervised framework that integrates exchange-based nutritional prescription into the OBEDIGITHUM outpatient obesity-care model. It also includes a retrospective observational analysis of anonymized routine-care data. The study was not a clinical trial or a comparative intervention study: no research intervention was assigned, there was no randomization, and there was no concurrent control group.

OBEDIGITHUM is a clinician-led, digitally enabled pathway developed by our group to support comprehensive obesity care. Before the first specialist consultation, it collects a structured multidimensional dataset including anthropometric variables, body composition, obesity history, previous weight-loss attempts, pharmacological treatment, lifestyle factors, eating behavior, physical activity, functional status, quality of life, adherence to a Mediterranean dietary pattern, sleep-apnoea risk, and clinically relevant dietary restrictions. Previously obtained laboratory data are used to assess metabolic, renal, and hepatic risk, including calculation of the Fibrosis-4 Index when indicated.

This pre-consultation process allows the initial medical encounter to focus on clinical interpretation, diagnostic integration, severity assessment, therapeutic prioritization, and individualized decision-making rather than on basic data collection. The nutritional prescription is developed within this broader diagnostic and therapeutic process rather than independently from it.

### 2.2. Retrospective Cohort Analysis

The retrospective analysis used anonymized routine-care data from 3928 unique patients (data cutoff 19 June 2026). Baseline weight values outside 30–300 kg were considered implausible, and the 6-month outcome was defined as the measurement closest to day 183 within a ±60-day window, after exclusion of implausible baseline-to-follow-up changes >50 kg and application of a one-record-per-patient rule. Patients without an eligible 6-month measurement were retained for denominator and baseline-selection comparisons; missing outcomes were not imputed. Baseline medication exposure was assessed only through free-text mentions available at baseline, which did not establish prescription, dispensing, dose, timing, duration, persistence, or adherence.

Continuous variables are summarized using means and standard deviations or medians and interquartile ranges, and categorical variables using counts and percentages. Baseline characteristics were compared descriptively between patients with and without an eligible 6-month weight, retaining variable-specific denominators and missing values. Standardized differences (eligible 6-month weight minus no eligible weight) summarize baseline differences in means or proportions, using variable-specific nonmissing observations. Weight change, total body-weight-loss categories, and the exploratory comparison according to baseline GLP-1 receptor-agonist mention are reported descriptively; no causal or comparative-effectiveness model was fitted. The present analysis did not measure adoption, fidelity, consultation time, workload, adherence, patient-reported usability, cost, or sustainability, which are therefore treated as implementation outcomes requiring prospective evaluation.

Secondary descriptive PREDIMED score analysis. Mediterranean dietary-pattern adherence was assessed using the 14-item PREDIMED score (Mediterranean Diet Adherence Screener, MEDAS), a validated instrument routinely recorded within OBEDIGITHUM [21]. Among 1575 patients with a baseline score, one follow-up score was selected as the available assessment closest to day 183 within days 183–365 (six months to one year). The lower-baseline subgroup was defined post hoc as all paired observations with baseline PREDIMED score ≤6, the empirical nearest-rank 25th percentile, including ties; this yielded 159/455 paired observations (34.9%), rather than exactly one quarter of the sample. Paired changes are summarized descriptively with fixed-seed bootstrap 95% confidence intervals (10,000 resamples); no *p* values or causal inferences are reported.

### 2.3. Dietcamb^®^ Exchange-Based Nutritional Method

Dietcamb^®^ was developed in 2006 by a multidisciplinary team comprising endocrinologists, dietitians/nutritionists, nurses, and information-technology professionals involved in the care of patients with obesity and cardiometabolic disease. It was conceived as a software-supported method for prescribing different types of clinical diets through a standardized exchange-based structure, rather than as a stand-alone nutrition application or a fixed diet restricted to obesity treatment [17,18].

The method builds on traditional food-exchange systems, in which substitutions preserve the overall nutritional structure of the diet while allowing flexibility in food selection [16]. Dietcamb^®^ extends this principle by linking individualized energy and macronutrient targets to functional food groups, exchange units, meal distribution, household measures, food lists, and practical patient-oriented materials. Within Dietcamb^®^, one exchange is defined as a standardized unit corresponding to 10 g of the predominant macronutrient within a given food group. Foods are classified into six functional groups—dairy products, protein foods, carbohydrates, vegetables, fruits, and fats—according to their predominant macronutrient contribution, relative composition, and energy density (Table 1). This classification is based on nutritional profile rather than conventional culinary categories. Thus, cured cheeses, nuts, and legumes are included within the protein group because of their relevant protein contribution.

Because foods contain more than one macronutrient, Dietcamb^®^ performs internal calculations to distribute the prescribed amounts of carbohydrate, protein, and fat across the different functional groups. Fruits and vegetables are allocated separately to ensure their inclusion within a nutritionally balanced dietary pattern and are not considered freely interchangeable with other carbohydrate-containing foods.

Foods within the same group are not completely interchangeable in unrestricted combinations. Although they share a predominant macronutrient, they may differ in energy density and secondary nutrient composition. Food lists are therefore subdivided according to their caloric and nutritional contribution, reducing the risk of systematic selection of higher-energy options and deviation from the prescribed energy target.

Average energy and macronutrient values were derived from food composition tables using foods commonly available and consumed in the Spanish dietary environment [22]. During development, weekly menus were constructed using different food combinations based on the same exchange distribution. This process was used to verify the nutritional coherence of the method in practical dietary patterns and informed successive refinements of the food lists, household measures, culinary equivalences, and example menus.

Dietcamb^®^ has been used and progressively refined for more than a decade in individual dietitian/nutritionist consultations. It can be adapted to different energy requirements, macronutrient distributions, clinical conditions, dietary restrictions, and therapeutic objectives. Its purpose is not merely to generate a menu, but to translate a nutritional prescription into a structured framework that patients can use to select foods and plan meals.

### 2.4. Integration of Dietcamb^®^ and Generation of the Individualized Prescription

The multidimensional information collected through OBEDIGITHUM informs the initial nutritional prescription generated by Dietcamb^®^. Basal metabolic rate is calculated using the Mifflin equation [23], after which a moderate energy deficit is applied according to age and sex: 500 kcal/day in men younger than 60 years, 400 kcal/day in men aged 60 years or older and in women younger than 60 years, and 300 kcal/day in women aged 60 years or older.

These values constitute initial rule-based targets and may be modified according to clinical condition, treatment objectives, previous dietary intake, body composition, functional status, and anticipated tolerance.

Macronutrient distribution is adapted to metabolic phenotype and body composition. Carbohydrate intake is assigned within a range of 40–50% of total energy according to the presence and severity of insulin resistance, metabolic syndrome, or type 2 diabetes.

Protein intake ranges from 20% to 30% of total energy and is adjusted according to indexed skeletal muscle mass and renal function: 20% when indexed skeletal muscle mass is >10 kg/m^2^ in men or >8 kg/m^2^ in women; 25% when values are 8–10 kg/m^2^ in men or 7–8 kg/m^2^ in women; 30% when values are <8 kg/m^2^ in men or <6.5 kg/m^2^ in women.

Fat intake is adjusted between 30% and 40% of total energy according to the carbohydrate and protein targets, prioritizing foods rich in monounsaturated fatty acids.

Once the energy and macronutrient targets have been established, Dietcamb^®^ distributes them across the six functional food groups and calculates the corresponding number of exchanges. These exchanges are then allocated across meals to generate a clinician-facing dashboard summarizing the daily prescription by food group and eating occasion.

Dietcamb^®^ automates only selected components of this process (Table 2). The final prescription remains clinician-supervised. The physician may modify the energy target, macronutrient distribution, meal number and timing, exchange allocation, food exclusions, and other practical aspects according to obesity phenotype, comorbidities, body composition, renal function, pharmacological treatment, eating pattern, food preferences, dietary restrictions, and lifestyle constraints.

Meal structure may also be adapted to treatment-related requirements. For example, patients receiving GLP-1 receptor agonists or other incretin-based therapies may require a more fractionated eating pattern to improve gastrointestinal tolerance and facilitate adequate protein and nutrient intake.

The pathway also incorporates predefined safety alerts. Eating-disorder risk is screened using the Eating Disorder Inventory, and clinically relevant depressive symptoms are assessed using the depression subscale of the Goldberg Anxiety and Depression Scale. The original instruments are described by Garner et al. [24] and Goldberg et al. [25]. High-risk results prompt individualized assessment before the standard nutritional pathway is implemented. Depending on the findings, evaluation by a clinician, dietitian/nutritionist, or mental-health professional may be required, and the prescription may need to be modified or deferred.

An estimated glomerular filtration rate below 50 mL/min/1.73 m^2^ similarly triggers individual review of protein intake and of the overall dietary prescription. Allergies, intolerances, coeliac disease, renal impairment, and other clinically relevant restrictions are managed through clinician-supervised adaptation rather than automatic exclusion rules.

### 2.5. Patient-Oriented Materials and Digital Delivery

Following clinical validation, the quantitative prescription is translated into patient-oriented materials comprising food-exchange lists, household culinary measures, visual and practical serving equivalences, food-substitution guidance, and example menus adapted to the prescribed exchange distribution.

The materials reduce the need for systematic food weighing by translating exchange units into familiar portions and household measures while preserving the prescribed energy and macronutrient structure. Example menus are not intended as closed or mandatory plans; they illustrate how different food combinations can be constructed from the same individualized prescription.

The food lists prioritize nutrient-dense options consistent with a Mediterranean dietary pattern and classify foods according to nutritional quality and energy density. They also indicate appropriate substitutions and identify higher-energy options that should not be selected systematically. When required, the materials can be adapted for allergies, intolerances, coeliac disease, renal impairment, or other dietary restrictions.

The validated prescription, dashboard, exchange lists, practical equivalences, and example menus are delivered through the OBEDIGITHUM patient portal after the initial consultation. The portal includes menus covering prescribed energy targets from 1200 to 2000 kcal. During the consultation, body-composition/bioimpedance and laboratory information are considered together with the clinical assessment to confirm individual energy requirements and select materials aligned with the final prescription. Patients subsequently work with these materials during structured online group sessions led by dietitians/nutritionists, with nursing participation within the broader pathway. These sessions address exchange interpretation, household measures, substitutions, energy-density differences, menu planning, eating behavior, physical activity, and practical application in daily life.

Patient-oriented materials and educational explanations have been progressively refined through routine clinical use and multidisciplinary review [18]. Difficulties identified during consultations and online sessions included misunderstanding of exchange equivalence, uncertainty regarding substitutions, insufficient clarity of household measures, and frequent selection of higher-energy options within the same group. Feedback from patients and healthcare professionals informed successive revisions of the food lists, visual equivalences, examples, menus, and educational explanations.

This iterative process reflects pragmatic, service-user-informed refinement rather than a formal co-design or patient-reported usability study.

### 2.6. Ethics and Data Governance

The retrospective analysis of fully anonymized routine-care data was approved by CEIm-FJD (05/24). The need for informed consent was waived for the retrospective analysis.

## 3. Results

### 3.1. Implementation Outputs of the NUTRI-OBEDIGITHUM Pathway

The implementation of Dietcamb^®^ within the OBEDIGITHUM workflow generated three main operational outputs: a clinician-facing prescription dashboard, patient-oriented dietary materials, and a structured educational pathway to support real-life use of the prescription.

First, the clinician-facing dashboard translates the individualized nutritional prescription into a standardized distribution of exchanges across food groups and meals. It provides a concise visual representation of the prescribed energy and macronutrient targets after their conversion into daily food exchanges, while allowing clinician-supervised adaptation to patient preferences, comorbidities, dietary restrictions, treatment context, and safety considerations. In routine practice, the dashboard supports consistency across professionals and facilitates delivery of a complete nutritional prescription during the first OBEDIGITHUM clinical visit.

Second, the prescription is transformed into patient-oriented materials, including food-exchange lists, household culinary measures, practical serving equivalents, and example menus adapted to the prescribed exchange distribution. These materials are designed to convert a quantitative prescription into real-life food choices without requiring systematic food weighing. Multiple food combinations can therefore be generated from the same exchange structure while preserving the intended energy target, macronutrient distribution, and food-group balance.

Food selection within these materials prioritizes nutrient-dense foods consistent with a Mediterranean dietary pattern, including vegetables, fruits, legumes, whole grains, nuts, fish, olive oil, and fermented dairy products, while limiting ultra-processed foods, refined sugars, and foods rich in saturated fat [26,27,28]. When clinically required, the lists and menus can be adapted to exclude allergens, gluten-containing foods, lactose-containing products, or other restricted components.

Third, the dietary prescription and patient-oriented materials are delivered through the OBEDIGITHUM patient portal after the initial consultation and are reinforced at a group visit one month later, during which patients review and learn how to use the individualized diet in daily life. The planned longitudinal pathway then includes five further group follow-up visits and two medical follow-up visits. Group sessions led by dietitians/nutritionists focus on understanding the exchange system, applying culinary equivalences, adapting menus, making food substitutions, and integrating the dietary plan into daily life. They also address broader lifestyle components, including physical activity and eating behavior, within the longitudinal OBEDIGITHUM care model. This structured contact sequence is intended to support dietary follow-up; adherence was not measured using a standardized instrument in the present study.

### 3.2. Process Evaluation and Service-User-Informed Refinements

Implementation of the pathway generated several process-related observations. The exchange-based structure allowed the nutritional prescription to be standardized without imposing a closed diet plan, and the use of household measures and practical equivalences reduced the need for systematic food weighing. Difficulties identified during routine consultations and educational sessions mainly concerned interpretation of exchange equivalence, selection of foods with higher energy density within the same group, and the need for clearer practical examples. These observations informed successive refinements of the food lists, household measures, example menus, and educational explanations.

The pathway therefore produced not only a prescriptive output but also an iterative implementation process in which patient-facing materials were progressively adapted according to practical barriers encountered by patients and professionals. This refinement was pragmatic and service-user-informed; it was not a formal co-design study and did not include standardized patient-reported usability, adherence, workload, fidelity, adoption, or time-saving measures.

### 3.3. Retrospective Cohort Flow

The source database contained 3928 unique patients. Of these, 3840 had a recorded baseline weight and 3784 had a plausible baseline weight within the prespecified 30–300 kg range. The 6-month window was observable for 3215 patients by the data cutoff, and a complete 6-month window was observable for 2811. An eligible 6-month weight was available for 929 patients, whereas 2286 target-reachable patients had no eligible measurement (Table 3).

Percentages use the explicit denominator shown in each row. Both the complete-window count (2811) and the eligible-weight count (929) are expressed relative to the 3215 patients whose 6-month window had opened; the eligible-weight row is not conditional on the preceding complete-window row. Thus, the eligible-weight proportion is 929/3215 (28.9%). The 6-month outcome was defined as the measurement closest to day 183 within a ±60-day window. Missing outcomes were not imputed.

### 3.4. Baseline Characteristics According to Outcome Availability

The analyzed cohort included 929 patients with an eligible 6-month weight. Their mean age was 53.7 ± 14.0 years, 655/929 (70.5%) were women, mean baseline weight was 98.5 ± 20.7 kg, and mean baseline body mass index was 35.9 ± 6.3 kg/m^2^. Among the 2286 patients whose 6-month window had opened but who lacked an eligible measurement, mean age was 51.8 ± 13.4 years, 1575/2285 (68.9%) were women, mean baseline weight was 99.0 ± 22.0 kg, and mean baseline body mass index was 35.9 ± 6.8 kg/m^2^. Variable-specific denominators were retained for BMI and recorded type 2 diabetes because of missing values (Table 4). Signed standardized differences are shown in Table 4; these descriptive comparisons do not exclude selection bias.

### 3.5. Baseline Medication Mentions

In the 929-patient outcome cohort, baseline free-text medication fields contained mentions of semaglutide in 90 patients (9.7%), liraglutide in 41 (4.4%), tirzepatide in 23 (2.5%), other GLP-1 receptor agonists in 7 (0.8%), and other anti-obesity drugs in 22 (2.4%). Categories could overlap. These data represent text mentions rather than validated medication exposure and do not establish prescription, dispensing, timing, dose, duration, persistence, or adherence.

PREDIMED score follow-up from six months to one year (exploratory) Of 1575 patients with a baseline PREDIMED score, 1318 had the start of the six-to-twelve-month window observable and 455 (34.5%) had a paired score in that window. Across the 455 paired observations, mean PREDIMED score changed from 7.41 at baseline to 8.00 at the selected follow-up assessment (median follow-up day 230). The descriptive mean change was +0.59 points (bootstrap 95% CI +0.44 to +0.75); 156/455 improved, 239/455 were unchanged, and 60/455 worsened.

**Figure 1:**
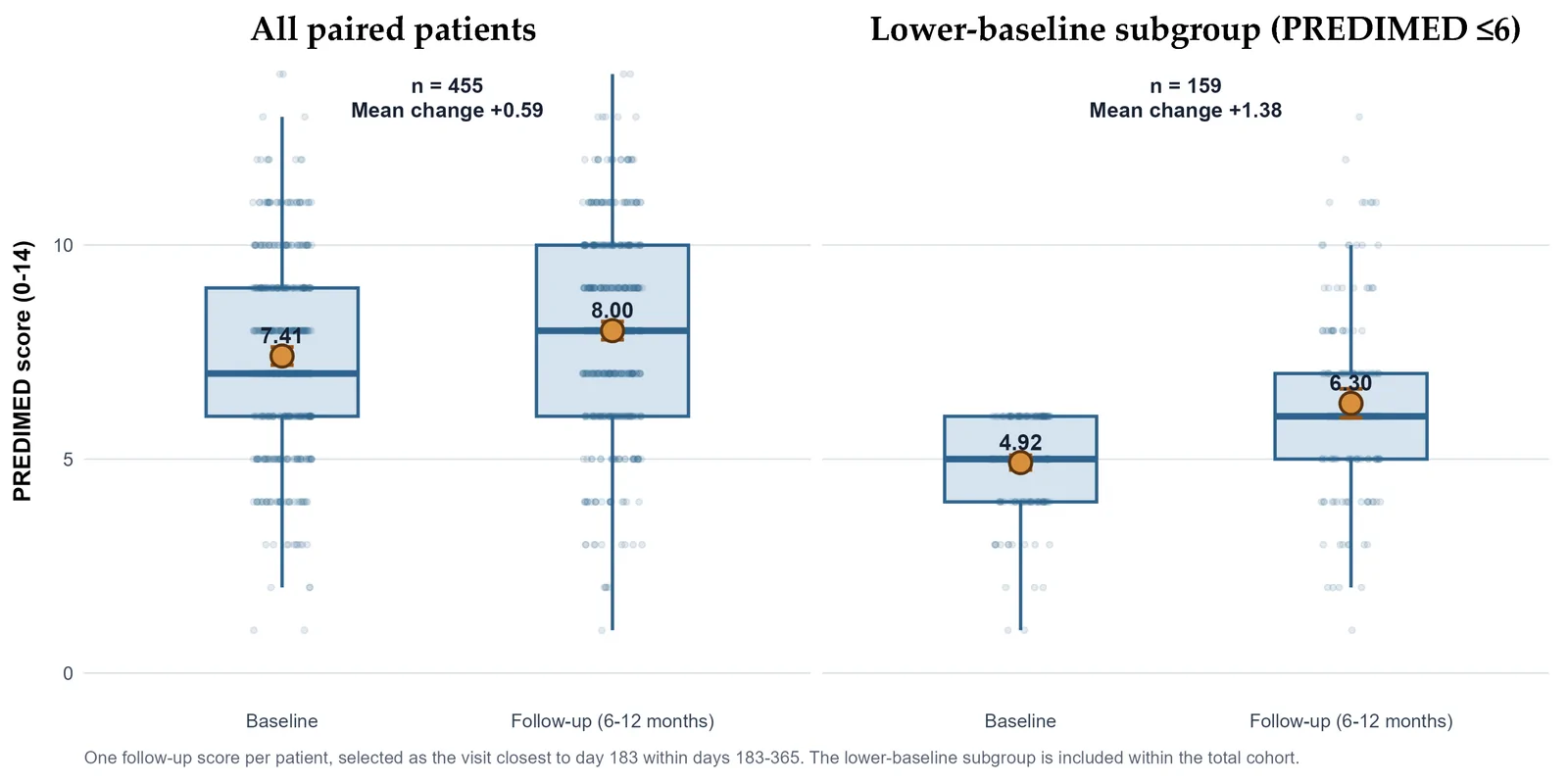
PREDIMED score at baseline and follow-up. Box plots and individual observations summarize the 14-point PREDIMED score at baseline and at follow-up (6–12 months); orange points and bars show group means and 95% t intervals. The total cohort includes 455 paired patients (mean change +0.59, bootstrap 95% CI +0.44 to +0.75); the exploratory lower-baseline subgroup (PREDIMED ≤ 6; *n* = 159, 34.9% of paired observations) is contained within that cohort and had mean change +1.38 (bootstrap 95% CI +1.10 to +1.67). One follow-up score per patient was selected as the visit nearest to day 183 within days 183–365. The analysis is selected complete-case and the score is not Dietcamb^®^-specific adherence.

In the exploratory lower-baseline subgroup (PREDIMED ≤ 6; 159/455 paired observations, 34.9%, including ties). Table 5 shows PREDIMED score changed from 4.92 to 6.30. The descriptive mean change was +1.38 points (bootstrap 95% CI +1.10 to +1.67) and the median change was +0.00; 75/159 improved, 81/159 were unchanged, and 3/159 worsened. The paired cohort and exploratory subgroup are shown in Figure 1.

### 3.6. Preliminary Real-World Weight Outcomes

Among the 929 patients with an eligible 6-month weight, mean body weight changed from 98.5 ± 20.7 kg at baseline to 94.2 ± 20.3 kg at follow-up. The descriptive paired change was −4.3 kg (95% CI −4.7 to −3.9). Median follow-up was 190 days [IQR 176–212], and median total body-weight loss was 3.4%. A total of 39.6% of patients achieved at least 5% total body-weight loss, 17.8% achieved at least 10%, and 6.4% achieved at least 15%.

These observations are based on patients with an eligible follow-up measurement and therefore represent selected complete-case trajectories. They are not estimates of the average outcome among all patients entering the pathway and cannot isolate the contribution of Dietcamb^®^, the broader OBEDIGITHUM pathway, pharmacotherapy, education, or other concurrent care.

### 3.7. Exploratory Comparison According to Baseline GLP-1RA Mention

Among patients with an eligible 6-month weight, 151 had a baseline GLP-1 receptor-agonist mention and 778 had no such mention. Median total body-weight loss was 4.0% [IQR 0.5–8.7] in the mention group and 3.2% [IQR 0.0–7.4] in the no-mention group. At least 5%, 10%, and 15% total body-weight loss were observed in 44.4%, 20.5%, and 6.6% of the mention group and in 38.7%, 17.2%, and 6.3% of the no-mention group, respectively (Table 6).

This stratification was exploratory and unadjusted. A medication-text mention does not establish treatment exposure, and the available records did not provide medication initiation date, dose, dispensing, duration, persistence, or adherence. Differences between groups should therefore not be attributed to GLP-1 receptor-agonist treatment or to the nutritional pathway.

## 4. Discussion

NUTRI-OBEDIGITHUM addresses the bottleneck identified in the Introduction: converting multidimensional obesity phenotyping into an individualized, clinician-supervised nutritional prescription within the time and staffing constraints of high-volume specialist care. Its main contribution is operational. The pathway combines two tools developed and progressively refined by our group: OBEDIGITHUM, which provides multidimensional phenotyping, clinical interpretation, and the digital infrastructure for longitudinal care [17,18], and Dietcamb^®^, which translates individualized energy and macronutrient targets into food exchanges, meal distribution, household measures, food lists, and example menus [19,20]. Their integration links the nutritional prescription to obesity phenotype, body composition, comorbidities, pharmacological treatment, eating behavior, dietary restrictions, and patient preferences, and makes it available from the first specialist encounter rather than after a wait for individual dietitian/nutritionist consultation.

To our knowledge, no published model has described a clinician-supervised, phenotype-linked exchange-based nutritional prescription delivered at this patient volume. Existing digital obesity-care programs are largely built around behavioral coaching, self-monitoring, or automated meal generation rather than an individualized prescription validated by a specialist [13,14,15]. For example, a recent controlled trial of a multicomponent digital therapy for obesity enrolled 246 patients across two centers [26]. That trial sample and the annual service population described here represent different denominators and do not provide a comparative assessment of scalability. NUTRI-OBEDIGITHUM was instead built to operate within four hospitals that together receive more than 2000 new patients with obesity each year, and its exchange-based structure was designed to support individualized prescription within this service. Whether expansion can occur without a proportional increase in dietitian/nutritionist staffing requires prospective workload and resource-use evaluation.

Dietcamb^®^ was originally developed for individual dietitian/nutritionist consultations and has been refined in that context for more than a decade [17,18]; its purpose is not to generate a menu, but to give patients a structured framework for selecting foods, making substitutions, and planning meals around defined nutritional targets. We hypothesize that allowing substitutions within an individualized nutritional structure may help patients accommodate changes in routine, food availability, and preferences and could support adherence over time. However, adherence to Dietcamb^®^ was not measured in this study; this hypothesis requires prospective evaluation using standardized measures.

The exchange-based prescription is generated and clinically validated during the initial specialist encounter, translated into patient-oriented materials, and delivered through the OBEDIGITHUM digital platform; patients then work with these materials during structured online group sessions led by dietitians/nutritionists. This real-world integration demonstrates that the pathway operates within routine clinical workflow, but it does not constitute a formal, patient-reported usability evaluation. Patient understanding, satisfaction, dietary autonomy, adherence, workload, fidelity, adoption, and implementation time should be assessed prospectively using standardized instruments.

The exchange-based structure provides a practical bridge between quantitative nutritional targets and everyday food choices [16]. Household measures, visual equivalences, food-substitution guidance, and food lists subdivided by energy density reduce reliance on systematic food weighing while limiting the risk of default selection toward higher-energy options within the same functional group [17,18]. Dietcamb^®^ automates selected calculations and generates an initial prescription, but the final plan remains clinician-supervised: energy intake, macronutrient distribution, meal structure, food exclusions, and safety considerations can be modified according to metabolic phenotype, indexed skeletal muscle mass, renal function, pharmacological treatment, and individual constraints. This balance of automation and clinical judgment differentiates the system from stand-alone digital tools built primarily for self-monitoring, calorie tracking, or automatic menu generation [14,15].

The model also seeks to preserve nutritional adequacy and metabolic safety rather than focusing exclusively on caloric restriction, which may be particularly relevant in patients receiving GLP-1 receptor agonists or other incretin-based therapies, in whom substantial reductions in food intake can be accompanied by nutritional challenges and loss of lean mass. These considerations are part of the prescription framework itself and should not be interpreted as evidence that the pathway prevents treatment-related adverse outcomes.

Food selection is based predominantly on a Mediterranean dietary pattern for its nutritional quality, cultural suitability, and compatibility with long-term cardiometabolic care [27,28,29], although Dietcamb^®^ is not restricted to this pattern and can accommodate different energy targets, macronutrient distributions, and clinical diets. In the Spanish healthcare context described in the Introduction, where demand for obesity care is high and dietitian/nutritionist capacity is limited [9,10,11], NUTRI-OBEDIGITHUM organizes advanced phenotypic assessment and individualized nutritional prescription within routine care; structured online education extends dietitian/nutritionist-supported education to groups but does not replace individual care for patients with greater nutritional, psychological, renal, dietary, or literacy-related complexity.

The quantitative findings reported here should be read against this operational contribution rather than as an effectiveness evaluation. At the broader service level, the previously reported OBE-DIGITHUM pathway described illustrative changes during implementation: annual patients assessed increased from 2731 to 3559, mean interactions per patient decreased from 17.8 to 7.1, in-person endocrinologist visits decreased from six to three per year, and mean per-patient costs for specialist visits and metabolic testing decreased from €511 to €242 and from €159 to €75, respectively [18]. These metrics describe the functioning of the integrated OBE-DIGITHUM service rather than a controlled evaluation of Dietcamb^®^ or an estimate of the resource contribution of its nutritional component.

The broader evidence base illustrates why the present study cannot separate the contribution of nutritional follow-up from medication or the other elements of obesity care: in a randomized trial, Wadden et al. varied lifestyle modification and pharmacotherapy across four treatment arms to examine the contribution of each component [30], whereas obesity-pharmacotherapy trials more commonly provide the same structured lifestyle intervention to active-treatment and placebo groups, preventing inference about whether lifestyle modification augments drug response [31]. Accordingly, the weight and PREDIMED trajectories reported here are indirect, convergent observations, not evidence of Dietcamb^®^-specific adherence or efficacy independent of pharmacotherapy.

Of the patients whose 6-month window had opened, only a minority contributed an eligible weight outcome (see Limitations), and the observed mean paired change of −4.3 kg and median total body-weight loss of 3.4% describe this selected complete-case group only; they do not estimate the average outcome of all patients entering the pathway. The PREDIMED score provides a descriptive outcome closer to the nutritional domain than weight, but it is likewise not a measure of Dietcamb^®^-specific adherence, and the larger mean change observed in the lower-baseline subgroup is compatible with regression to the mean rather than differential effectiveness. The exploratory comparison by baseline GLP-1 receptor-agonist mention should be read the same way: free-text mentions do not establish medication exposure, dose, or duration, and the observed differences between groups should not be attributed to medication or to the nutritional pathway.

Overall, NUTRI-OBEDIGITHUM provides an operational framework through which an individualized exchange-based nutritional prescription can be generated, clinically validated, and delivered within routine specialist obesity care. The present study documents the clinical workflow and selected routine-care observations, but does not establish scalability, implementation effectiveness, adherence, or cost-effectiveness. Formal prospective evaluation of these outcomes is the necessary next step.

## 5. Limitations

This article provides a methodological and operational description of the pathway and does not assess its clinical effectiveness or its independent contribution within the broader OBEDIGITHUM model.

The retrospective weight analysis was uncontrolled and restricted to patients with an eligible follow-up measurement. The 929 analyzed patients represented 28.9% of the 3215 patients whose 6-month window had opened, creating potential selection, survivorship, and ascertainment bias. Missing outcomes were not imputed, and the observed trajectories should not be generalized to all patients entering the pathway.

Patient understanding, perceived usability, satisfaction, adherence, and dietary autonomy have not yet been evaluated using standardized instruments. These outcomes may vary according to health literacy, digital literacy, emotional status, cognitive burden, clinical complexity, and access to technology.

The pathway does not eliminate the need for individual nutritional care. Patients with eating-disorder risk, severe emotional symptoms, renal impairment, complex dietary restrictions, or relevant literacy barriers may require individualized assessment and follow-up. The screening procedures and clinician-supervised alerts are intended to identify these situations.

Patient- and professional-informed refinement of the materials occurred pragmatically through routine clinical use rather than through a formal co-design protocol. Future studies should evaluate patient experience, comprehension, adherence, nutritional adequacy, NUTRI-specific professional workload, implementation consistency, the proportion of patients requiring additional individual support, and resource use or cost-effectiveness attributable to the nutritional component. Such studies should document prescription delivery and session attendance, dietary-pattern change, and validated pharmacotherapy exposure, with a prespecified comparator when the objective is to estimate the contribution of the nutritional component.

Medication information was obtained from baseline free-text mentions and therefore did not provide validated exposure data. The analysis could not determine treatment initiation, dose, dispensing, duration, persistence, adherence, or changes during follow-up.

Finally, the pathway was developed within a specific Spanish healthcare network and dietary context. Its transferability may depend on healthcare organization, professional roles, digital infrastructure, availability of dietitians/nutritionists and nurses, and local food culture. Evaluation in other settings will be required to establish broader applicability.

## 6. Conclusions

NUTRI-OBEDIGITHUM represents an operational framework that incorporates individualized, exchange-based nutritional prescription into specialized longitudinal obesity care. Its main contribution is to link multidimensional patient characterization to a structured, flexible, and actionable nutritional intervention from the first clinical encounter.

By combining structure with flexibility, the approach can be adapted to the clinical heterogeneity of people living with obesity. Its real-world reach, scalability, implementation effectiveness, and contribution to clinical outcomes require prospective evaluation.

The framework addresses a relevant operational challenge in Spain: delivering individualized nutritional prescription within routine obesity care despite limited dietitian/nutritionist capacity.

The preliminary weight trajectories reported here are descriptive findings from a selected, uncontrolled complete-case cohort and should not be interpreted as evidence of independent pathway efficacy. Future evaluations should assess implementation effectiveness, patient understanding, dietary autonomy, adherence, nutritional adequacy, safety, NUTRI-specific professional workload and cost, and generalizability across healthcare settings.

Taken together, these elements describe NUTRI-OBEDIGITHUM as an operational approach to delivering individualized nutritional prescription within routine obesity care in the participating hospitals. Its scalability and transferability require prospective evaluation.

## Figures and Tables

**Table 1:** Food exchanges and nutrient content per exchange.

Code	Food Group	Nutrient Content per Exchange
1DA	Dairy	One exchange provides 10 g of carbohydrate plus approximately 7.5 g of protein, with fat content varying according to whether the product is skimmed, semi-skimmed, or whole. It is also rich in calcium.
1PR	Protein	One exchange provides 10 g of protein and a variable amount and type of fat.
1CH	Carbohydrates	One exchange provides 10 g of carbohydrate, with variable amounts of fiber and plant protein.
1VE	Vegetables	One exchange provides 10 g of carbohydrate and/or protein, in addition to a high content of fiber, vitamins, minerals, and antioxidants.
1FR	Fruits	One exchange provides 10 g of carbohydrate, in addition to a high content of fiber, vitamins, minerals, and antioxidants.
1FA	Fats	One exchange provides 10 g of fat, with the type of fat varying according to the food item.

Functional food groups used in Dietcamb^®^. One exchange is based on 10 g of the predominant macronutrient; foods are categorized by nutritional profile and caloric density. Abbreviations: DA, dairy; PR, protein; CH, carbohydrates; VE, vegetables; FR, fruits; FA, fats.

**Table 2:** Automated and clinician-supervised components of the Dietcamb^®^ prescription process.

Component	Rule-Supported Output	Clinician Input
Resting energy expenditure	Mifflin equation	Validation if clinically needed
Energy deficit	300–500 kcal according to sex/age rule	Adjustment in selected cases
Carbohydrate	40–50% according to insulin resistance/metabolic syndrome/diabetes	Clinical validation
Protein %	20–30% according to GFR and skeletal muscle mass index	Clinical validation
Fat %	30–40%, preferably monounsaturated-rich fatty acids	Adjusted according to carbohydrate/protein targets
Meal number/distribution	Not automatically fixed	Adapted to habits, preferences, and medication
Food exclusions	Not automatically fixed	Allergy, intolerance, celiac disease, or clinical restriction
Eating disorder risk	Safety alert	Individual review recommended

Automated rules generate initial targets and alerts; clinicians validate and individualize the final prescription. Abbreviations: GFR, glomerular filtration rate.

**Table 3:** Retrospective cohort flow.

Step	*n*	Denominator, N	*n*/N (%)
Unique patients in source cohort	3928	—	—
Patients with recorded baseline weight	3840	3928	97.8%
Patients with plausible baseline weight (30–300 kg)	3784	3840	98.5%
Baseline-weight cohort with 6-month window observable	3215	3784	85.0%
Baseline-weight cohort with complete 6-month window observable	2811	3215	87.4%
Patients with eligible 6-month weight	929	3215	28.9%

**Table 4:** Baseline characteristics according to 6-month outcome availability.

Characteristic	Eligible 6-Month Weight (*n* = 929)	Window Opened, No Eligible Weight (*n* = 2286)	Missing Values, Eligible/No Eligible Weight	Standardized Difference
Age, years, mean (SD)	53.7 (14.0)	51.8 (13.4)	0/0	0.143
Female sex, *n*/N (%)	655/929 (70.5%)	1575/2285 (68.9%)	0/1	0.034
Baseline weight, kg, mean (SD)	98.5 (20.7)	99.0 (22.0)	0/0	−0.028
Baseline BMI, kg/m^2^, mean (SD)	35.9 (6.3); *n* = 914	35.9 (6.8); *n* = 2230	15/56	−0.001
Type 2 diabetes recorded, *n*/N (%)	96/874 (11.0%)	213/2137 (10.0%)	55/149	0.033
Baseline GLP-1RA mention, *n*/N (%)	151/929 (16.3%)	302/2286 (13.2%)	0/0	0.086

BMI, body mass index; GLP-1RA, glucagon-like peptide-1 receptor agonist. Standardized differences compare patients with an eligible weight against those without: the difference in means divided by the square root of the average group variance for continuous variables, or the difference in proportions divided by the square root of the average p(1 − p) for binary variables. Positive values indicate higher values in the eligible-weight group. Values were calculated before rounding the displayed summaries and are reported to three decimal places. Denominators exclude missing observations for each variable. Medication status was based on baseline free-text mentions and does not validate treatment exposure.

**Table 5:** Change in 14-point PREDIMED score from baseline to the selected assessment between six months and one year.

Analysis Set	*n*	Baseline Mean	Selected 6–12-Month Mean	Mean Change (Bootstrap 95% CI)	Median Change	Improved/Unchanged/Worsened
All paired observations	455	7.41	8.00	+0.59 (+0.44 to +0.75)	+0.00	156/239/60
Exploratory lower-baseline subgroup (PREDIMED ≤ 6; ties included)	159	4.92	6.30	+1.38 (+1.10 to +1.67)	+0.00	75/81/3

**Table 6:** Exploratory stratification by baseline GLP-1RA mention.

Baseline Medication-Text Category	*n*	Median TBWL, % [IQR]	≥5% TBWL	≥10% TBWL	≥15% TBWL
GLP-1RA mention	151	4.0 [0.5–8.7]	67 (44.4%)	31 (20.5%)	10 (6.6%)
No GLP-1RA mention	778	3.2 [0.0–7.4]	301 (38.7%)	134 (17.2%)	49 (6.3%)

TBWL, total body-weight loss; GLP-1RA, glucagon-like peptide-1 receptor agonist. Medication categories were based on baseline free-text mentions, do not validate treatment exposure, and are presented as exploratory and unadjusted.

## Data Availability

The original contributions presented in this study are included in the article. Further inquiries can be directed to the corresponding author.
